# CRED: A Deep Residual Network of Convolutional and Recurrent Units for Earthquake Signal Detection

**DOI:** 10.1038/s41598-019-45748-1

**Published:** 2019-07-16

**Authors:** S. Mostafa Mousavi, Weiqiang Zhu, Yixiao Sheng, Gregory C. Beroza

**Affiliations:** Department of Geophysics, Stanford University, Stanford, CA United States

**Keywords:** Seismology, Geophysics, Seismology

## Abstract

Earthquake signal detection is at the core of observational seismology. A good detection algorithm should be sensitive to small and weak events with a variety of waveform shapes, robust to background noise and non-earthquake signals, and efficient for processing large data volumes. Here, we introduce the Cnn-Rnn Earthquake Detector (CRED), a detector based on deep neural networks. CRED uses a combination of convolutional layers and bi-directional long-short-term memory units in a residual structure. It learns the time-frequency characteristics of the dominant phases in an earthquake signal from three component data recorded on individual stations. We train the network using 500,000 seismograms (250k associated with tectonic earthquakes and 250k identified as noise) recorded in Northern California. The robustness of the trained model with respect to the noise level and non-earthquake signals is shown by applying it to a set of semi-synthetic signals. We also apply the model to one month of continuous data recorded at Central Arkansas to demonstrate its efficiency, generalization, and sensitivity. Our model is able to detect more than 800 microearthquakes as small as −1.3 ML induced during hydraulic fracturing far away than the training region. We compare the performance of the model with the STA/LTA, template matching, and FAST algorithms. Our results indicate an efficient and reliable performance of CRED. This framework holds great promise for lowering the detection threshold while minimizing false positive detection rates.

## Introduction

During the past 10 years, there has been an enormous increase in the volume of data being generated by the seismological community. Each year, more than 50 terabytes of seismic data are archived at the Incorporated Research Institutions for Seismology (IRIS) alone. The massive amount of data highlights the need for more efficient and powerful tools for data processing and analyses. The main challenge is the efficient extraction of as much useful information as possible from these large datasets. This is where rapidly evolving machine learning (ML) approaches have the potential to ply a key role^1–4^. One of the first stages that observational seismologists need to meet this challenge is in the processing of continuous data to detect earthquake signals. Among a large variety of detection methods developed in past few decdes, STA/LTA^5^ and template matching^6,7^ are the most commonly used algorithms. While STA/LTA is generalized and efficient, its sensitivity to time-varying background noise and lack of sensitivity to small events, false positives, and events recorded shortly after each other make it less than optimal for robust and sensitive detection. Although the high sensitivity of cross-correlation improves the detection threshold of template matching, the requirement of prior knowledge of templates and multiple cross-correlation procedures make it less general and inefficient for real-time processing of large seismic data volumes. Although more advanced algorithms such as Fingerprint And Similarity Thresholding (FAST)^8^ using Locality-Sensetive Hashing^9^ can improve the efficiency of the similarity search, the outputs are in that case limited to repeated signals.

Shallow fully connected Neural Networks (NN) were among the first ML methods used for the earthquake signal detection^10,11^. NN receive a feature vector, x (a sparse representation of seismic data), as input and transform it through a series of hidden layers to predict the desired outputs, y, in the output layer. Each hidden layer is made up of a set of neurons, where each neuron is fully connected to all neurons in the adjacent layers, and where neurons in a single layer function completely independently and do not share any connections. Non-linear activation functions inside neurons make learning complex x-y relations possible through an optimization process. To learn these relations more deeply and to build a more complex model requires exponentially more units in each layer or alternatively many layers with few units in each layer (a deep neural net). Fully connected layers, as in standard neural networks, don’t scale well to large input vectors and require careful initiation, feature engineering, optimization, and regularization to prevent vanishing/exploding gradients^12^ and overfitting.

During the recent renewed interest in ML applications among seismologists, some studies have revisited the detection problem^13–15^. These studies presented successful and promising performances of deep convolutional-neural-networks (CNN) for robust and efficient detection of parts of earthquake signals. In addition to fully connected layers, CNN’s include convolutional layers and pooling layers. A convolutional layer consists of a set of learnable filters (or kernels). Every filter has a small receptive field (connected to a small region of the layer before it). It applies a convolution operation to the input and produces an activation map that gives the responses of that filter at every spatial position. Hence, as the input volume is passing through the convolutional layers the network automatically learns the low/mid/high-level features in data. This eliminates the need for heavy pre-processing and hand engineering of features. Moreover, it reduces the number of free parameters, allowing the network to be deeper with fewer parameters. Pooling layers combine the outputs of multiple neurons at one layer into a single neuron in the next layer, hence it can reduce the dimension of learned features as data is fed forward.

In this paper, we formulate the detection problem as a sequence-to-sequence learning^16^ where a time series of inputs are mapped to a time series of probability outputs and separate predictions are made for each individual sample. Recurrent neural networks (RNN) were designed for processing sequential data like seismograms. They have internal state (memory) and can share features learned across the different positions within a time series^17^. Hence, they can learn the dynamic temporal relations within a time sequence. In this study, we use bi-directional long-short-term memory (LSTM) as our RNN units and employ a residual-learning framework to make deeper learning feasible. In the residual learning framework, the network will learn the residual functions instead of original mapping functions. This makes their optimization easier, keeps the training error in the deeper layer the same as for the shallower ones, and by doing so makes it possible to train a deeper network. Deeper networks allow learning higher-level features and building more complex models. We demonstrate the performance of the network using both semi-synthetic and real data.

## Results

### The network architecture

**Figure 1:**
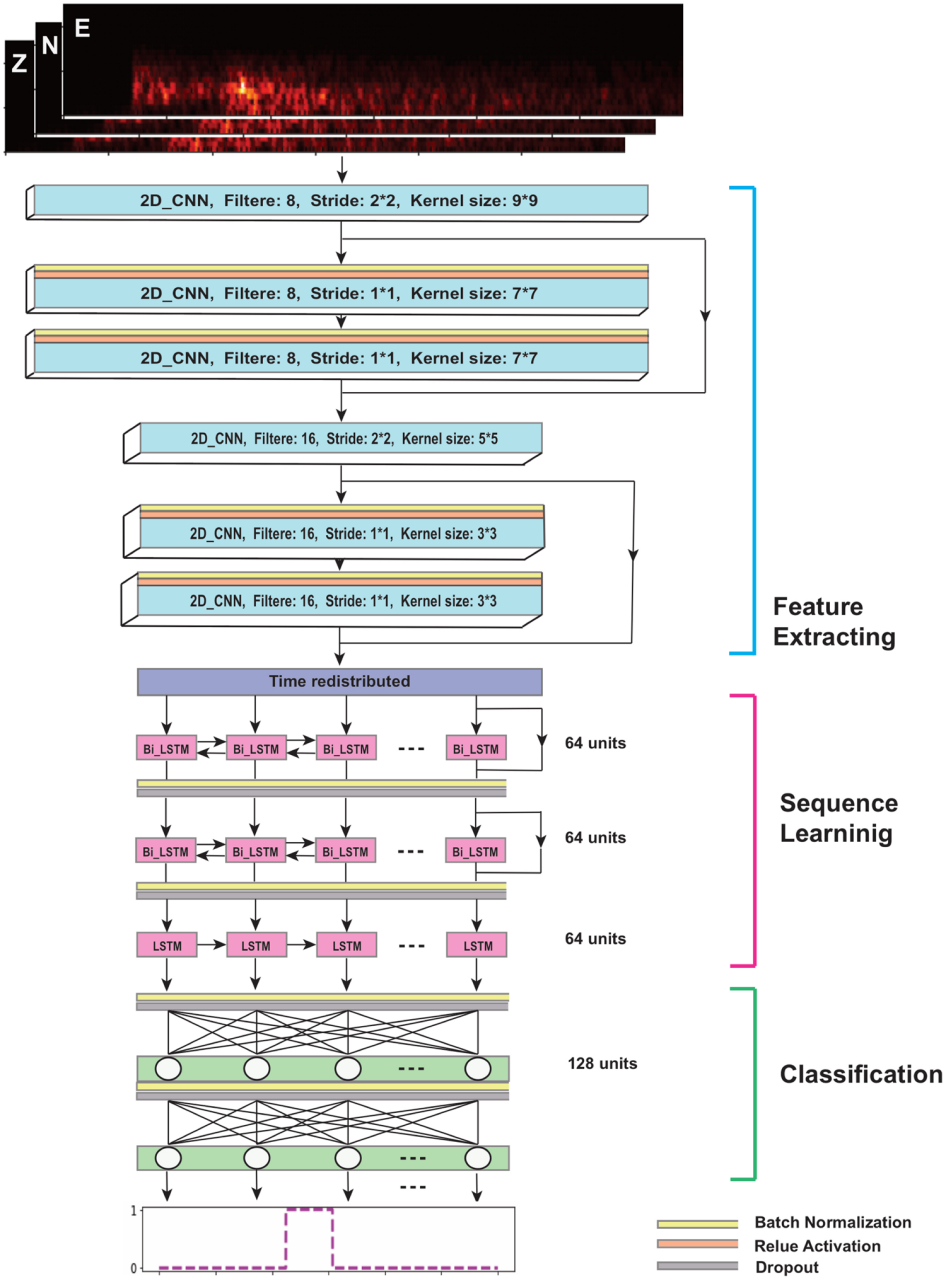
The architecture of our proposed deep neuralnetwork.

The architecture of our proposed network is presented in Fig. 1. We use three types of layers: convolutional, recurrent, and fully connected, in a residual structure.

Inputs into the network are spectrograms of three component seismograms. Our previous studies^18,19^ showed that time-frequency transforms can improve event detection by better characterizing seismic signals. The first few blocks (blue layers) consist of mainly convolutional layers. Convolutional layers in the residual block are preceded by a batch normalization (BN) and an activation layer. BN layers normalize the activation of a convolution layer during the training, which accelerates the training and has some regularization characteristics that help prevent overfitting. These layers will automatically learn and extract features from the input spectrograms so there is no need for feature engineering or complicated pre-processing. As the network gets deeper, these layers extract higher-level (i.e., finer scale) features.

To maximize the feature extraction we increased the number of 2D convolutional layers by arranging them into residual blocks based on the improved structure presented by He *et al*.^20^. This is done by providing shortcuts that make it possible to train a deeper network without degradation. Every two residual blocks are preceded by an individual layer with a stride of 2 that halves the feature map size. This decreases the feature map size with depth and down-samples the feature volume to a more sparse representation. The number of filters in every three layers is doubled to preserve the time complexity per layer. The down-sampling speeds the training of recurrent layers in the following section.

After 2D convolutional layers, the feature volume is redistributed into a sequence and passes into two residual blocks of bidirectional LSTMs. These layers learn and model the sequential pattern of data. Since in real time seismic acquisition, samples of an earthquake signal are recorded/represented with time increasing from left-to-right, we add one additional unidirectional LSTM layer to the end of the recurrent section. Finally, the last two layers of the network are fully connected layers that perform the high-level reasoning and map the learned sequence model from the last step to the desired output classes. We used a sigmoid binary activation function in the last layer of the network, and the output is a vector of predicted probabilities for each sample to contain an earthquake signal.

Overall the network consists of 12 layers and has 256,000 trainable parameters. It takes advantage of three different types of layers within an efficient structure. Design of the network with shortcuts in a residual learning structure makes it possible to train the network with an acceptably low error rate. This is an end-to-end learning framework that can characterize the seismic data with high precision. It is designed to be extensible and has the potential to fulfill many applications in automating seismic data processing.

### Data

**Figure 2:**
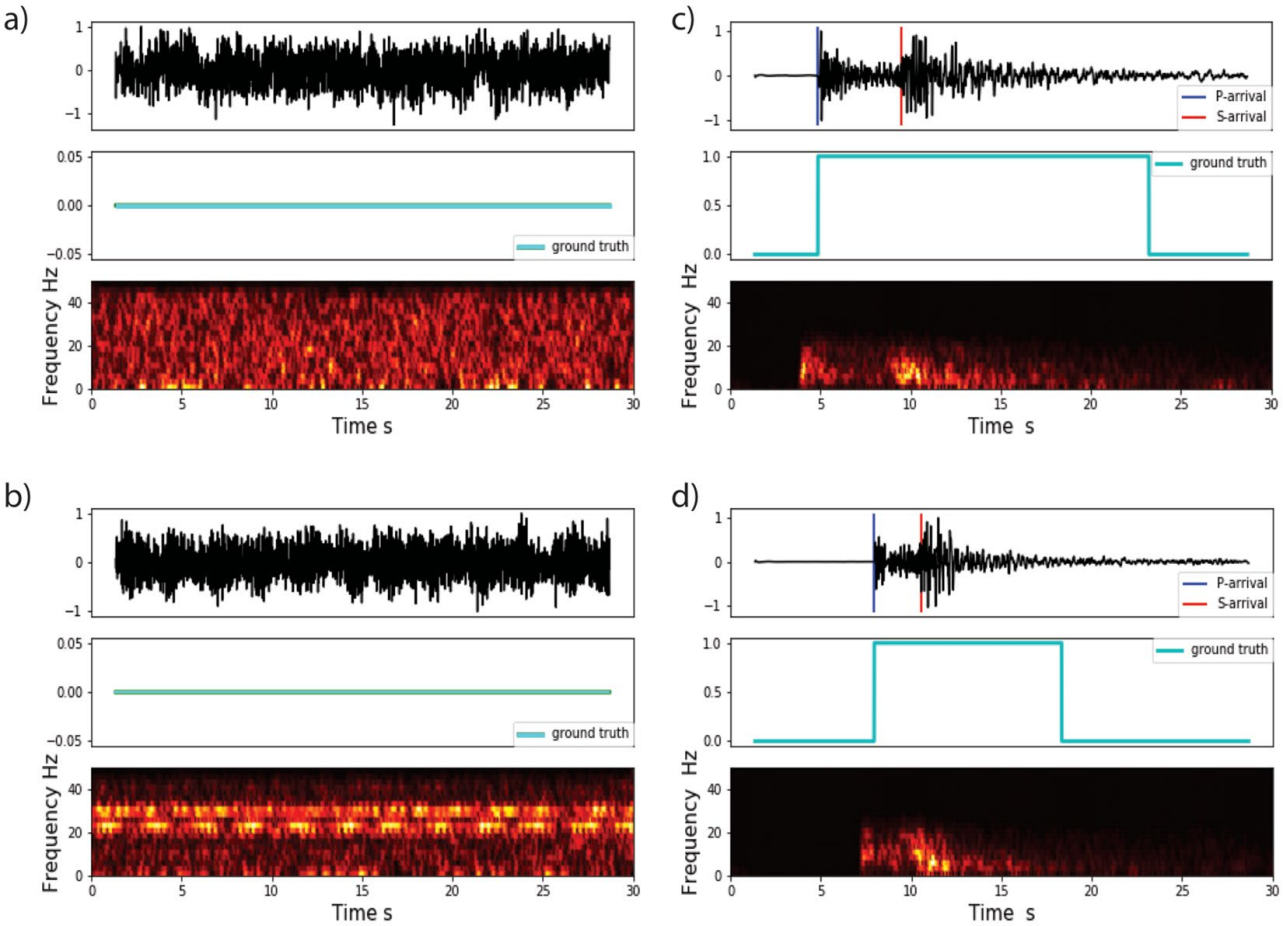
Examples of seismogram, label vector, and associated spectrogram (short time Fourier transform, STFT) for vertical components of two sample noises (**a**,**b**) and two earthquake samples (**c**,**d**).

We use 550,000 30-second 3-component seismograms recorded by 889 broadband and short-period stations in North California for the training of the network and its validation. 50% of these seismograms are associated with earthquakes occurring from January 1987 to December 2017. The metadata used for labeling the *P*-wave and *S*-wave arrival times are manual picks provided by the Northern California Earthquake Data Center. Another half of the data set consists of seismic noise recorded by the same network stations. The noise samples contain a variety of ambient and non-ambient noises Fig. 2. We randomly sampled 30-second noise seismograms obtained from time spans between the cataloged events. We then performed a simple quality check of the ratio of maximum over the median amplitude in each window to eliminate those noise traces that might contain uncatalogued events. To further ensure no contamination of the noise samples by small events below the background noise level, we used a de-signaling algorithm^21^ on the remaining traces. This algorithm removes the anomalous spectral features associated with earthquake signals.

We detrended all the traces by removing the mean, band-pass filtered between 1 and 45 Hz, resampled at 100 HZ, and normalized. We used the Short Time Fourier Transform to construct spectrograms. For ground truth, we generated one binary label vector with the same length as spectrograms. In the label vector, we set the value of corresponding samples from the P arrival to P + 3(S − P) to 1 and the rest to 0 representing the probabilities of existence of earthquake signal Fig. 2. This range captures the dominant spectral energy of P and shear waves, which characterize the earthquake signals in the time-frequency domain.

### Training and testing of the model

We randomly split the dataset into training (80%), evaluation (10%), and test (10%) sets. We trained the network on one Tesla V100-PCIE GPU. We used binary cross-entropy as the loss function and the ADAM algorithm^22^ for optimization. We completed the training in 62 epochs, and found that the validation accuracy did not improve in the last 20 epochs. The final training and validation accuracies are 99.33% and 99.24% respectively while the losses are 0.02 and 0.03. The mean absolute errors are 0.01 and 0.009 for the training set and validation set respectively. These values are measured from a point-by-point comparison of the model predictions with the ground truth.

**Figure 3:**
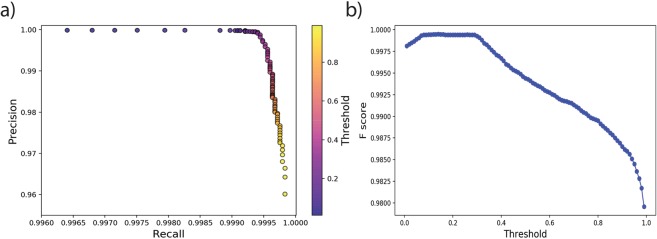
Precision-recall curve (**a**) and the F-score as a function of threshold values (**b**).

**Figure 4:**
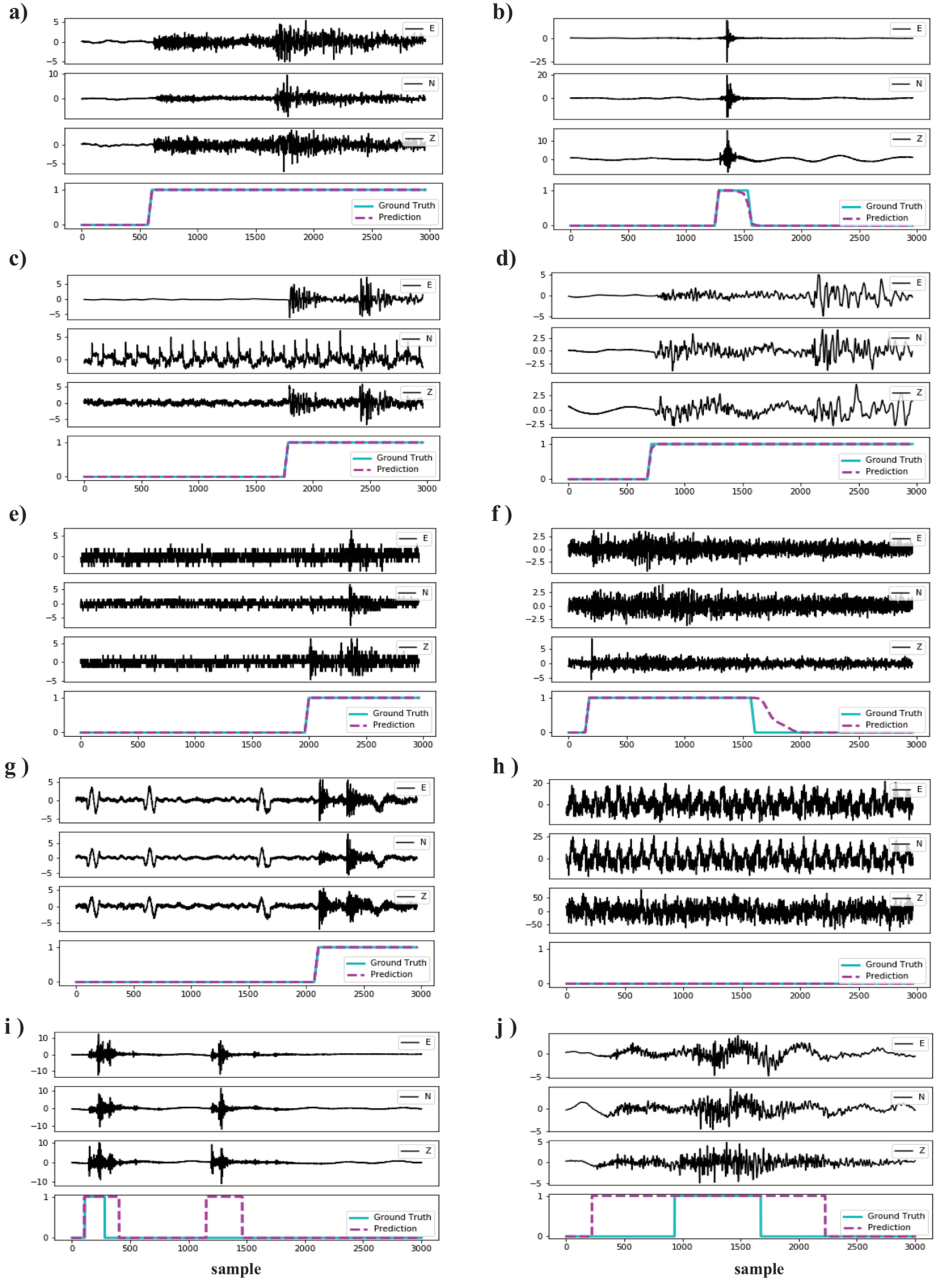
Results of applying the trained network on a few samples in the test set.

To evaluate the performance of the model for detection, we used 50,000 test samples. After selecting a decision threshold value (tr) for output probabilities, we can calculate several parameters for evaluating the performance of the model. We use precision, recall, and F-score as evaluation metrics. Precision is defined as the fraction of predictions that are accurate, Recall is defined as the fraction of instances that are accurately predicted, and the F-score (the harmonic mean of precision and recall) combines these two parameters to eliminate effects of unbalanced sample size for different classes:

1 $$Precision=\frac{TR(tr)}{TP(tr)+FP(tr)}.$$

2 $$Recall=\frac{TP(tr)}{TP(tr)+FN(tr)}.$$

3 $$F-Score=\frac{2\times Precision\times Recall}{Precision\,+Recall}.$$

where, TP denotes true positives, FP denotes false positives, and FN are false negatives. In a perfect classifier *TP* = 1, and *FP* = 0. The precision and recall are calculated using different threshold values to check the effect of different threshold selection on the detection results Fig. 3. As can be seen from Fig. 4, regardless of the threshold choice, the model test results in a precision higher than 96% and a recall above 99%. Threshold values between 0.1 and 0.3, however, lead to the maximum F-score of 99.95. In Table 1 we report the confusion matrix for the threshold value of 0.11.

**Table 1:** Confusion Matrix for Threshold = 0.11.

Detected as ->	Earthquake	Noise
Earthquake	25,230	2
Noise	7	24,761

From Fig. 4 we see that the network was able to generalize its learning and predict the entire duration of earthquake signals (including S coda) with high precision no matter if the event is large Fig. 4a or small Fig. 4b, local Fig. 4b or teleseismic Fig. 4d, recorded on an instrument with a malfunctioning channel Fig. 4c, recorded on old stations Fig. 4e, contaminated with high background noise level Fig. 4f, or not earthquake signals Fig. 4g. Since the predictions are performed for each sample individually, the network does not require the full length of a signal to detect an earthquake. This is important for real-time processing. In Fig. 4i we see that it was able to detect an event that was missing from the catalog we used for the labeling, or to correct the mislabeled P arrival and estimated end of coda in Fig. 4j, and by doing so, in these instances it improved upon “ground truth”.

### Sensitivity to the noise level

**Figure 5:**
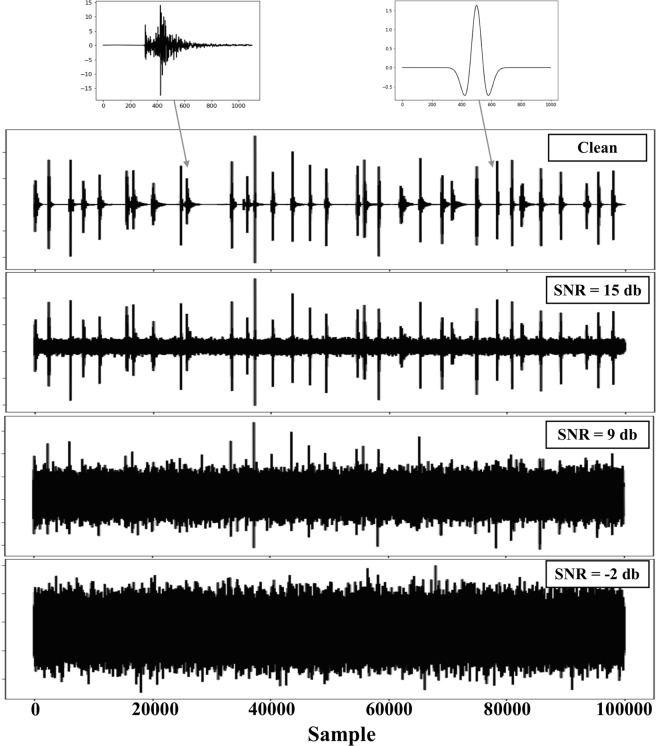
1000 seconds of the generated syntheticseismogram (vertical component) with the different noise level. The insets atthe top present one example of the earthquake and non-earthquake signals.

To explore the performance sensitivity of the CRED to the background noise level and compare it with the other popular algorithms, we performed a test using semi-synthetic data. We selected 500 events with high-SNR from the test set. We visually checked each seismogram prior to the test. We also generated 500 Ricker wavelets with random width and scaled amplitudes to represent impulsive non-earthquake signals. Earthquake and non-earthquake signals were then randomly assembled to generate 8.4 hours of continuous data. Next, we added 23 different levels of Gaussian noise to the continuous waveforms in a way that result in 23 realizations of data with SNRs ranging from −2 dB to 20 dB. SNR is measured as

$$10\times {\mathrm{log}}_{10}({[{S}_{A}/{N}_{A}]}^{2})\,$$

where *S*_*A*_ and *N*_*A*_ are peak amplitudes of signal and noise respectively. A portion of the generated synthetic data is presented in Fig. 5.

**Figure 6:**
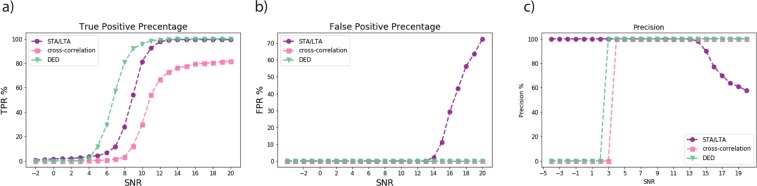
Comparison of the performance of the proposedmethod (CRED) with STA/LTA and template matching on semi-synthetic data withdifferent noise levels.

We applied the CRED, STA/LTA, and template matching on the synthetic data. For the template matching two templates were used. The detection threshold for each of these algorithms was tuned carefully to maximize the precision. The results are presented in Fig. 6.

As can be seen from the Fig. 6, CRED had superior performance compared to the two other methods. It detects 100% events with SNR >= 12 db with 0 false positives for all SNRs. At SNR of 7 db, CRED detected 80% of events while the detection rates for STA/LTA and template matching at this level were 27% and 3% respectively. In general CRED can detect far more events at low SNRs with a very low false positive rate. Template matching also resulted in no false positives but it had the lowest detection rate (81.8% at 20 db) due to the fact that most of the events were not located nearby or recorded on the same station. On the other hand STA/LTA resulted in the highest false positive rate (72.6% at 20 db). This is due to the sensitivity of its characteristic function to the abrupt amplitude changes, which causes a low precision for the STA/LTA at high SNRs Fig. 6c. The superior performance of CRED in noisy condition could be due to its reliance on spectral contents of the signal rather than the waveform. Although our results indicate lower sensitivity of CRED to the background noise level compared to other traditional methods, noise still affects the detection performance. Hence, applying a seismic denoising algorithms^23–28^ prior to the detection can improve the results.

### Application to central U.S

A good model that does not overfit the data set used for the training, should generalize well to other data sets. To test this characteristic of our detector and the model obtained using the North California data set, we apply CRED to continuous data recorded in Guy-Greenbrier, Arkansas, during the 2010–2011 sequence^29,30^. Several earthquake catalogs exist for this sequence based on different detection methods: ANSS^29^ and Ogwari *et al*.^31^ based on STA/LTA, Huang and Beroza^32^ based on template matching, and Yoon *et al*.^33^ based on FAST. August 2010 is the overlapping time period in these catalogs, hence we made a unified catalog for this period combining information of all the above catalogs containing 3788 events. Most of these events are microearthquakes with local magnitudes ranging from −1.3 to 0.5 associated with hydraulic fracturing or wastewater injection. Events are located within a 2 km area at 2 to 4 km depths. Template matching^32^ has the highest detection rate (3732), FAST^33^ detected 3266 of the events, and studies based on STA/LTA^29,31^ detected 23 and 24 events respectively.

**Figure 7:**
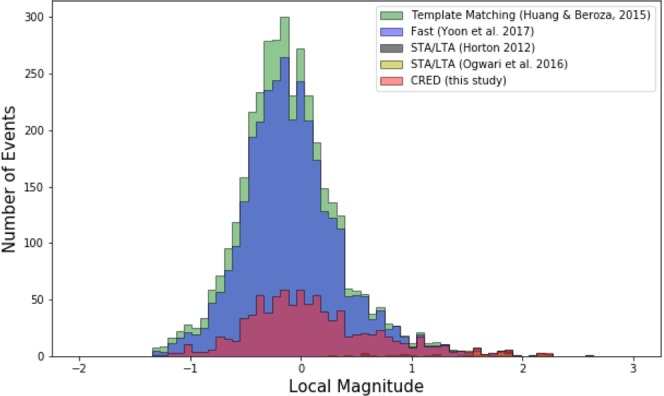
Magnitude-frequency distribution of detected events using different algorithms.

We process one month of continuous data recorded at station WHAR during August, 2010. This is the common station used in these studies. Detecting induced microearthquakes in different regions and at the local scale in the presence of high noise levels is a challenging task for a detection algorithm. We applied the CRED algorithm using a moving window of 30 seconds. The total processing time including transferring three-channel data into STFT and applying the model was 1 hour and 9 minutes on a laptop with a 2.7 GHz Intel Core i7 processor and 16 GB of memory. Our model detected 1102 events. Of these, 680 events were already in the catalog. We visually checked the remaining detections and verified 77 of them as new events and 345 of them as false positives. This leads to a detection precision of 69%. Detected events by CRED range from magnitude −1.2 to 2.6 Fig. 7. Lowering the detection threshold can lead to detection of more cataloged events; however, it would also result in more false positives.

**Figure 8:**
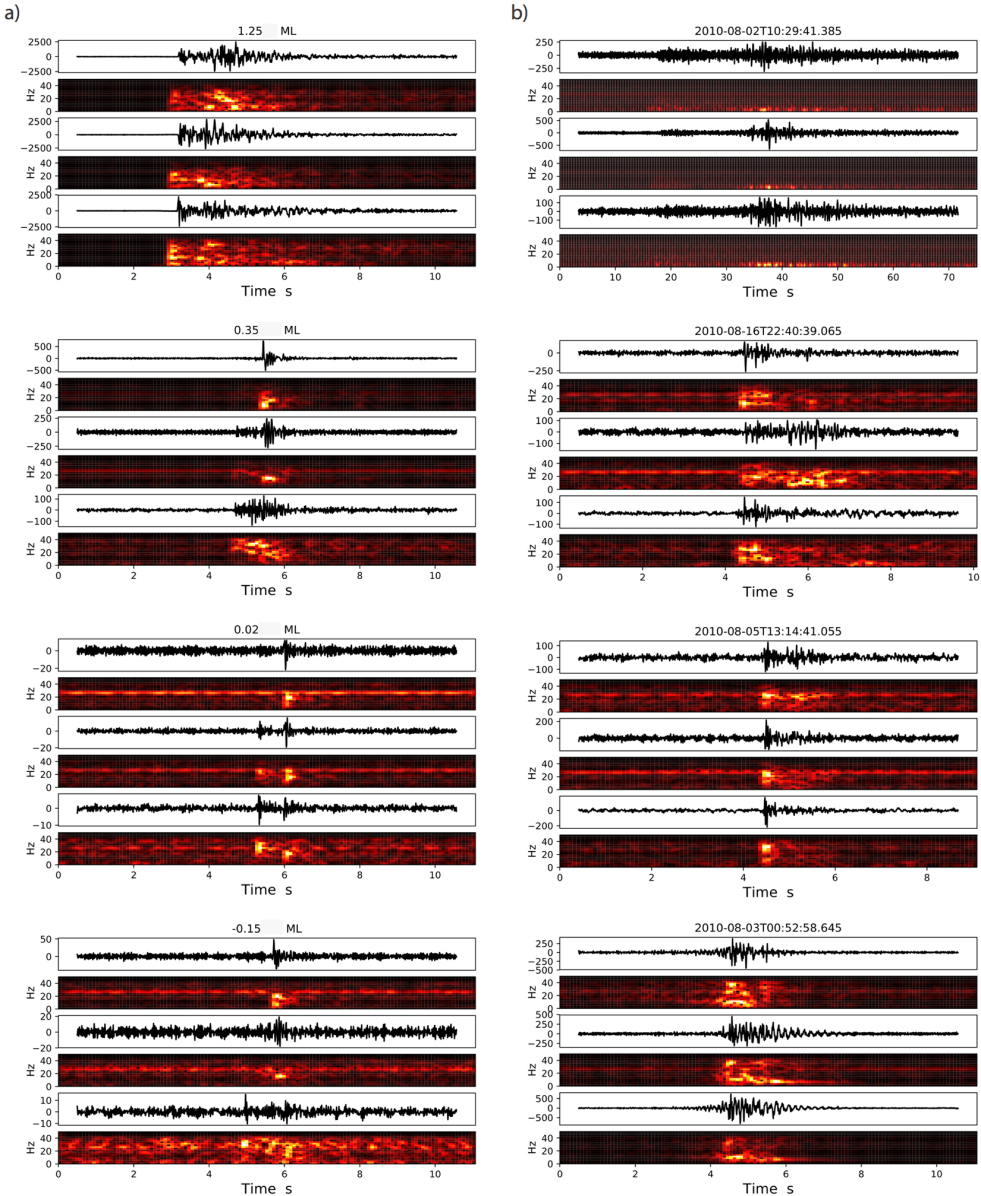
Examples of detected events in Arkansas. Left column (**a**) are events that have been detected by previous studies and existed in our unified catalog. The magnitude of each event is taken from Yoonet al. (2017) and is listed on top of each plot. The right column (**b**) shows some events newly detected by CRED. The detection time of each event is shownon the plot headers.

A few examples of events detected by CRED are presented in Fig. 8 Almost all of the events larger than *M*_*L*_ 1.0 have been detected. The detection extends to lower magnitudes and includes a variety of SNRs and waveforms. Magnitude calculation of newly detected events is beyond the scope of this paper; however, by comparing the amplitudes and durations of these events with the waveforms of some of the cataloged events we estimate 0.1 < *M*_*L*_ < 0.4 for these events.

## Discussion

Reliable earthquake signal detection is at the core of observational earthquake seismology. While improving the sensitivity and robustness of current algorithms is still an active field of research, improving the efficiency has received increased attention in the recent years due to a significant increase in data volumes. In this paper we explored the application of a deep residual network of convolutional and recurrent units for earthquake signal detection. Convolutional layers are powerful tools for automatic extraction of sparse features from seismograms. Recurrent units can learn sequential characteristics of seismic data and provide robust models. Here we used LSTM, a powerful type of recurrent network to search for earthquake signals in the time-frequency domain.

We designed the network with a residual structure to prevent degradation and reach a higher accuracy with a deeper learning. The residual-learning structure of the network makes a very-deep end-to-end learning of seismic data feasible. The proposed network, can learn the dynamic time-frequency characteristics of earthquake signals and build a generalized model using a modest-sized training set. Using time-frequency representation of seismic data and the sparsity of learned features by the CNN layers make the model robust to the noise. Sensitivity tests revealed the superior performance of CRED in the presence of high noise levels compared with both STA/LTA and template matching.

**Figure 9:**
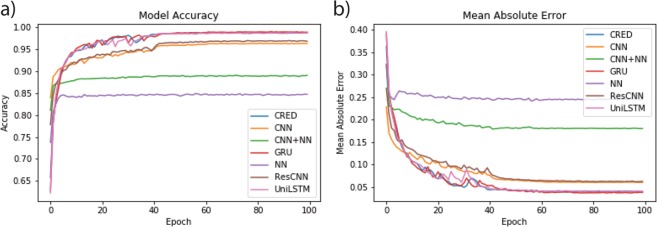
Comparison of different network architectures on a test data set. Accuracy (**a**) and mean absolute error (**b**) for the proposed architecture of this study (CRED), a network containing both convolutional and fully connected layers (CNN + NN), a network similar to the CRED with bidirectional GRU units instead of LSTM (GRU), a traditional fully connected neural network (NN), a network containing both convolutional and fully connected layers in residual structure(ResCNN + NN), and a network similar to the CRED with unidirectional LSTM (UniLSTM).

To compare the performance of our proposed network architecture with other common neural networks, we performed a separate experiment training six different networks - our proposed residual CNN + RNN + NN (CRED) network, a network containing both convolutional and fully connected layers (CNN + NN), a network similar to the CRED with bidirectional GRU units instead of LSTM (GRU), a traditional fully connected neural network (NN), a network containing both convolutional and fully connected layers in residual structure (ResCNN + NN), and a network similar to the CRED with unidirectional LSTM (UniLSTM). We trained all these networks using 1000 samples and measured their relative performance in terms of classification accuracy and the mean absolute error Fig. 9. The fully connected network (NN) tends to have the lowest accuracy and the highest error. Using a combination of convolutional and fully connected layers (CNN + NN) results in higher accuracy and lower error. Recasting the CNN + NN network in a residual learning structure improves the performance, however it is still lower than the CRED (residual CNN + RNN + NN). Using unidirectional LSTM instead of bidirectional LSTM slightly decreases the performance in earlier epochs (<40) however the overall results are very close and using bidirectional LSTM does not have a significant effect. We have also tested replacing the bidirectional LSTM units in CRED with bidirectional GRU units. GRU or gated recurrent units^34^ are a type of simplified LSTM known to perform better on some problems; however, we did not find any significant improvement in our application. Both networks (LSTM and GRU) resulted in very similar accuracy and error.

**Figure 10:**
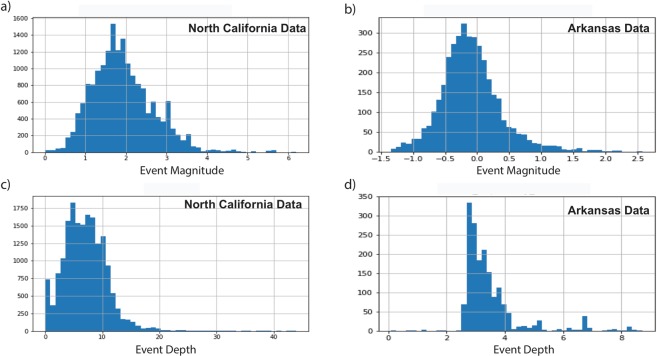
Statisticsof the North California Data set used for the training and the Arkansas dataset.

The learned model generalizes well to seismic data recorded in other regions. We trained the network using local and regional waveforms recorded in Northern California. The events mainly have tectonic origins, range in magnitude from M = 0–5, with most events around M 2 Fig. 10, and epicentral distances of mostly around 50 km. The model was tested on detecting microseismic events in Central Arkansas, with substantially different crustal structure, much lower magnitude range (mainly −1.0 to 0.5 M), shorter epicentral distance (within 3 km), and generally shallower depths. This represents an exceptionally challenging test of the method, but our model performed acceptably by detecting 3 orders of magnitude more events compared with STA/LTA. Although CRED’s detection rate was 30% of template matching and FAST, unlike template matching, no templates were used in its training. Moreover, it ran more than 100 times faster than FAST (non-parallel version) and resulted in lower false positive rates (31%) compared to FAST (55%). CRED resulted in 69% detection precision while the precision of detection was 45% for FAST^33^. Detection of new events missed by all previous studies indicates an inherent limitation of similarity-search based methods in addition to their high computational costs.

Here we used only 250 k waveforms of earthquakes with limited parameter distribution for training. Retraining the network with a much larger data set with a higher variability of earthquake signals with different hypocentral parameters and magnitude ranges should improve the performance. Moreover, a recursive approach can be adapted by the inclusion of events detected in the first round of applying the model into retraining and updating the initial model. Including more reliable labels through a secondary cross-validation step or incorporating different types of non-earthquake signals recorded on seismic stations are other steps that could be implemented to improve the method further.

A potential application of CRED is in real-time monitoring of seismic activity for hazard mitigation in tectonically active regions or areas of induced seismicity. Microseismic monitoring and earthquake early warning are other areas where CRED could be used. This framework can provide fast and reliable event detection and is easily scalable to large N (many sensor) or large T (long duration) data. Reprocessing large volumes of archived data can potentially lead to new insights into a wide variety of earthquake phenomena.

In this paper we present a Cnn-Rnn Earthquake Detector (CRED), which is a network that combines convolutional and recurrent units for deep residual learning of the time-frequency characteristics of earthquake signals. This framework is capable of detecting seismic events with high efficiency and precision. The learned model has low sensitivity to the background noise level, can generalize well to other regions with different seismicity characteristics, and outperforms STA/LTA in terms of sensitivity and template matching in terms of efficiency. Application of our method is fast and once the network is trained it can be applied to a stream of seismic data in real time. The architecture is flexible and can be easily scaled. False positive rates are minimal due to the high-resolution modeling of earthquake signals based on their spectral structure.

## Methods

### Sequential learning

**Figure 11:**
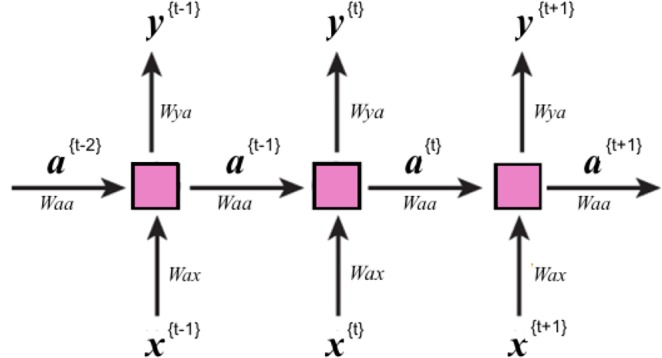
Schematic of RNN for many-to-many elements. Internal states, a, are passing through the network and at each position used for calculating the prediction y.

The RNN performs sequential learning by retaining relations among inputs while training itself Fig. 11.

A nonlinear function of the weighted sum of input at time *t*, *x*^(*t*)^, and the previous state (or learned activation in the previous time step), *a*^(*t*−1)^, is used to compute the current state, *a*^(*t*)^, and predict the output at time *t*, *y*^(*t*)^.

4 $${a}^{(t)}=gt({W}_{aa}{a}^{(t-1)}+{W}_{ax}{x}^{(t)}+{b}_{a}).$$

5 $${y}^{(t)}=gs({W}_{ya}{a}^{(t)}+{b}_{y}),$$

where the *W*’s and *b*’s are associated weights and bias terms, and *gt* and *gs* are Tanh and Sigmoid activation functions:

6 $$gt(z)=\frac{{e}^{z}-{e}^{-z}}{{e}^{z}+{e}^{-z}}.$$

7 $$gs(z)=\frac{1}{1+{e}^{-z}}.$$

**Figure 12:**
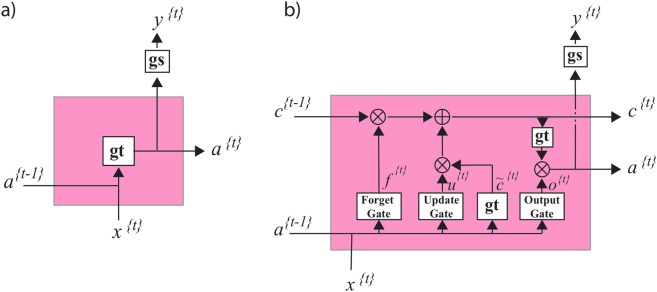
(**a**) A closer look at a standard RNN unitin the previous figure. Inside each unit, a Tanh activation function, gt, is applied to the internal statesfrom the previous position and the input of current position to obtain theinternal state, a, at currentposition. This state then will be passed to the next position and also will beused to predict the output of the current position, y, using either a Sigmoid or SoftMax function, gs. (**b**) An LSTM unit.These units in addition to the long-term memory, a, have a short-term memory c.

This basic RNN unit Fig. 12a, however, is not effective for learning long sequences due to the vanishing/exploding gradient problem. Some specific types of RNN such as long-short-term memory (LSTM) are commonly used to reduce the vanishing/exploding gradient problem and make application of deeper networks feasible.

An LSTM^35^ unit has an internal memory cell, c, which is simply added to the processed input. This greatly reduces the multiplicative effect of small gradients. The time dependence and effects of previous inputs are controlled by a forget gate, which determines what states are remembered or forgotten. Two other gates, the update gate, and an output gate are also featured in LSTM cells Fig. 12b.

8 $${\tilde{c}}^{\{t\}}=gt({W}_{c}{a}^{\{t-1\}}+{W}_{c}{x}^{\{t\}}+{b}_{c}).$$

9 $${u}^{\{t\}}=gs({W}_{u}{a}^{\{t-1\}}+{W}_{u}{x}^{\{t\}}+{b}_{u}).$$

10 $${f}^{\{t\}}=gs({W}_{f}{a}^{\{t-1\}}+{W}_{f}{x}^{\{t\}}+{b}_{f}).$$

11 $${o}^{\{t\}}=gs({W}_{o}{a}^{\{t-1\}}+{W}_{o}{x}^{\{t\}}+{b}_{o}),$$

where ${\tilde{c}}^{\{t\}}$ is a candidate value for replacing the memory, ${u}^{\{t\}}$ is an update gate, ${f}^{\{t\}}$ is a forget gate and ${o}^{\{t\}}$ is the output gate. The value of the memory cell at each time step will be set using the candidate value at current step, ${\tilde{c}}^{\{t\}}$, and previous value, ${c}^{\{t-1\}}$, based on update and forget gates:

12 $${c}^{\{t\}}={u}^{\{t\}}\,\ast \,{\tilde{c}}^{\{t\}}+{f}^{\{t\}}\,\ast \,{c}^{\{t-1\}},$$

where * is element-wise multiplication. The final state at time *t*, ${a}^{\{t\}}$, is obtained based on output gate and the value of memory cell:

13 $${a}^{\{t\}}={o}^{\{t\}}\,\ast \,gt({c}^{\{t\}}).$$

**Figure 13:**
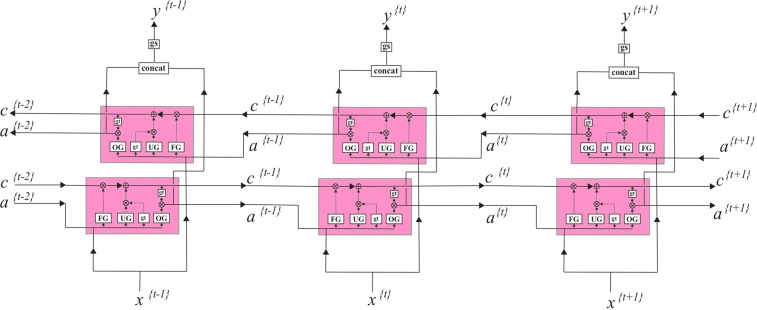
Bidirectional LSTM layer.

These LSTM units can be connected to each other in a row to form one hidden layer of the RNN Fig. 13. To learn the temporal pattern both from left to right and right to left, we can simply concatenate two of these layers with opposite directionality to form a bidirectional^36^ RNN layer Fig. 13. This structure allows the networks to have both backward and forward information about the sequence at every time step.

### Residual learning

In an end-to-end deep network, the “levels” of learned features can be enriched by the number of stacked layers (depth). For that reason, the number of layers (depth of a network) plays a crucial role in allowing the network to learn more high-level features and build more complex models. There are, however, two main problems that occur in the training of very deep networks (where “deep” is usually taken to mean more than 15 layers): (1) vanishing/exploding gradients and (2) degradation (saturation and degradation of accuracy). The vanishing/exploding gradients problem occurs when the network cannot learn because the gradients of the network’s output with respect to the parameters in the early layers become extremely small or large^12^, and can be largely addressed by normalized initialization. An effective solution for the latter was proposed by He *et al*.^20^ by introducing the residual learning framework.

**Figure 14:**
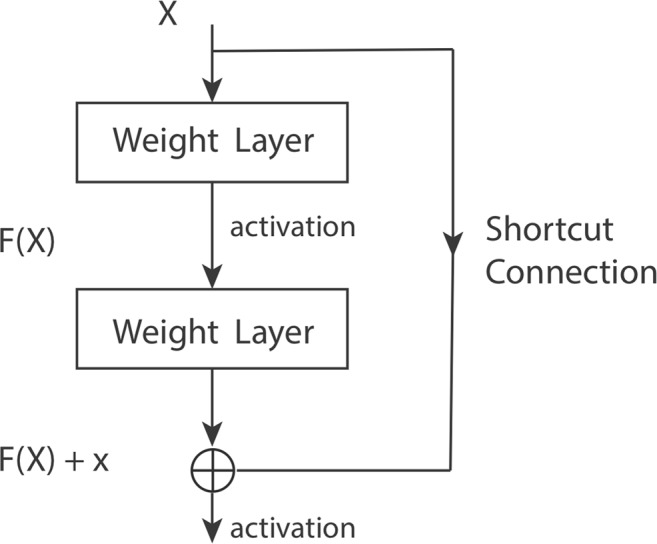
A building block in Residual learning.

In a standard deep network, every few stacked layers basically learn a nonlinear underlying function, H, that maps the input to the first of these layers, x, to the output of the final layer, H(x). In the residual learning framework, the original mapping is recast into F(x) + x. Each few stacked layers (a residual block) rather than directly learning H(x), learns the residual functions, F(x): = H(x) − x. Since optimization of the residual mapping is easier than for the original unreferenced mapping, this can help to keep the training error in the deeper layer the same as for the shallower ones, and by doing so make it possible to train a deeper network. Residual learning blocks can be realized by feed-forward neural networks with “shortcut connections” Fig. 14 that skip one or more layers. The shortcut connections simply perform identity mapping, and their outputs are added to the outputs of the stacked layers Fig. 14. These identity shortcut connections add neither extra parameters nor computational complexity to the problem.

In residual learning a building block is defined as:

14 $$y=F(x,\{{W}_{i}\})+x.$$

Here x and y are the input and output vectors of the layers considered. The function $F(x,\{{W}_{i}\})$, represents the residual mapping to be learned. For the example in Fig. 1 that has two layers, $F={W}_{2\sigma }({W}_{1x})$, in which $\sigma$ denotes the activation function and the biases are omitted t simplifying notations. The operation F + x is performed by a shortcut connection and element-wise addition. As noted above, the shortcut connections introduce neither extra parameters nor computation complexity.
